# Fine Structure in Multi-Phase Zr_8_Ni_21_-Zr_7_Ni_10_-Zr_2_Ni_7_ Alloy Revealed by Transmission Electron Microscope

**DOI:** 10.3390/ma8074618

**Published:** 2015-07-22

**Authors:** Haoting Shen, Leonid A. Bendersky, Kwo Young, Jean Nei

**Affiliations:** 1 BASF-Ovonic, 2983 Waterview Drive, Rochester Hills, MI 48309, USA; E-Mails: haoting.shen@nist.gov (H.S.); jean.nei@basf.com (J.N.); 2 Material Measurement Laboratory, National Institute of Standards and Technology, Gaithersburg, MD 20899, USA; E-Mail: leoben@nist.gov

**Keywords:** intermetallics, nanocrystalline structure, microstructure, planar faults, scanning electron microscopy, transmission electron microscopy

## Abstract

The microstructure of an annealed alloy with a Zr_8_Ni_21_ composition was studied by both scanning electron microscopy (SEM) and transmission electron microscopy (TEM). The presence of three phases, Zr_8_Ni_21_, Zr_2_Ni_7_, and Zr_7_Ni_10_, was confirmed by SEM/X-ray energy dispersive spectroscopy compositional mapping and TEM electron diffraction. Distribution of the phases and their morphology can be linked to a multi-phase structure formed by a sequence of reactions: (1) L → Zr_2_Ni_7_ + L’; (2) peritectic Zr_2_Ni_7_ + L’ → Zr_2_Ni_7_ + Zr_8_Ni_21_ + L”; (3) eutectic L” → Zr_8_Ni_21_ + Zr_7_Ni_10_. The effect of annealing at 960 °C, which was intended to convert a cast structure into a single-phase Zr_8_Ni_21_ structure, was only moderate and the resulting alloy was still multi-phased. TEM and crystallographic analysis of the Zr_2_Ni_7_ phase show a high density of planar (001) defects that were explained as low-energy boundaries between rotational variants and stacking faults. The crystallographic features arise from the pseudo-hexagonal structure of Zr_2_Ni_7_. This highly defective Zr_2_Ni_7_ phase was identified as the source of the broad X-ray diffraction peaks at around 38.4° and 44.6° when a Cu-K was used as the radiation source.

## 1. Introduction

Zr-based AB_2_ metal hydride (MH) alloys are considered as an alternative for the misch metal-based AB_5_ MH alloys currently used as the active material in the negative electrode of the nickel/metal hydride (Ni/MH) rechargeable batteries that are used in consumer and hybrid electric vehicle applications. The AB_2_ MH alloys offer higher capacities [1,2,3], higher flexibility in composition design [4,5,6,7], and are exempt from the market price volatility of rare earth metals. Beside the Laves phase-based pseudo-binary AB_2_, different Zr-Ni intermetallics (with or without substitution) are also of great interest for the improvement in both capacity and high-rate dischargeability (HRD) of the MH alloys. For example, ZrNi_5_ [8], Zr_2_Ni_7_ [9], ZrNi_3_ [10], Zr_8_Ni_21_ [11,12], ZrNi_2_ [13,14], Zr_7_Ni_10_ [15,16,17], Zr_9_Ni_11_ [18], ZrNi [19,20], and Zr_2_Ni [21] with different partial substitutions were studied as MH alloys for the potential application in Ni/MH batteries. Out of these Zr-Ni intermetallic alloys, Zr_8_Ni_21_ is very important since its stoichiometry is ideal for room temperature applications, such as the Ni/MH battery. It is not normally seen as a secondary phase in the multi-phase Zr-based MH alloys due to its peritectic origin in the phase diagram. The Zr_8_Ni_21_ phase is not stable and is seldom observed in partially substituted Zr_8_Ni_19_*X*_2_ alloys [11]. The undoped Zr_8_Ni_21_ shows a gas-solid storage of 0.63% mass fraction hydrogen at 30 °C and an electrochemical discharge capacity of 136 mAh·g^−1^ [12]. In this study, X-ray diffraction (XRD) of both as-cast and annealed (960 °C for 8 h) Zr_8_Ni_21_ samples consistently shows the presence of broad peaks around 38.4° and 44.6°, which could not be reconciled with XRD and scanning electron microscopy (SEM) results (Figure 2a,b in [11]). In the same paper, the broad peaks from XRD analysis were suspected to be a result of the fine microcrystal mixtures from the ZrNi and ZrNi_5_ phases found by SEM/X-ray energy dispersive spectroscopy (EDS) analysis. Similar broad peaks were also found in a paper by Ruiz and his coworkers for a Zr_8_Ni_21_ sample annealed at 1000 °C for 30 days, but were not identified [22]. Since the peaks cannot be explained by International Centre of Diffraction Data [23], and the broadness was not consistent with other peaks, the presence of one or more additional phases was suspected. Transmission Electron Microscopy (TEM) applied together with other chemical-sensitive techniques (EDS, electron energy-loss spectroscopy, *etc*.) is capable of providing both structural and chemical information at the sub-atomic level [24,25,26]. Crystal structure, chemical composition, or atomic bonding can be easily characterized [27,28,29]. In this paper, we investigated the same alloy by advanced TEM technique with the goal to identify the origin of the broad peaks and find the suspected minor phase(s). The full understanding of the phase components in the Zr_8_Ni_21_ base alloy is very important for the future development of multi-phase Zr-based MH alloy with various substitutions for electrochemical applications.

## 2. Experimental Section

The ingot sample was prepared by arc melting 10 g of Zr and Ni mixture with the stoichiometric ratio of 8:21, respectively, under a continuous argon flow with a non-consumable tungsten electrode and a water-cooled copper tray. Before melting the Zr and Ni mixture, a piece of titanium used as oxygen getter underwent a few melting-cooling cycles to reduce the residual oxygen concentration in the system. The ingot was re-melted and flipped over a few times to ensure uniformity in chemical composition. The ingot was annealed at 960 °C for 8 h in an argon environment. The average chemical composition of the sample was examined with a Varian Liberty 100 inductively coupled plasma (ICP) system (Analytical West, Inc., Corona, CA, USA). A Rigaku Miniflex XRD (Rigaku Corporation, Tokyo, Japan) was used to study the phases of the sample. JEOL JSM7100 field emission SEM (JEOL Ltd., Tokyo, Japan) with EDS capability was used to study the sample’s microstructure and compositional distribution by collecting EDS mapping data from polished surface of the sample. For TEM measurements, samples were thinned by a mechanical polish followed by an ion milling. FEI Titan 80-300 TEM/STEM (FEI, Inc., Hillsboro, OR, USA) was used to study the microstructure of the alloy samples.

## 3. Results and Discussion

### 3.1. Phases, Microstructure, and Formation Path

**Figure 1:**
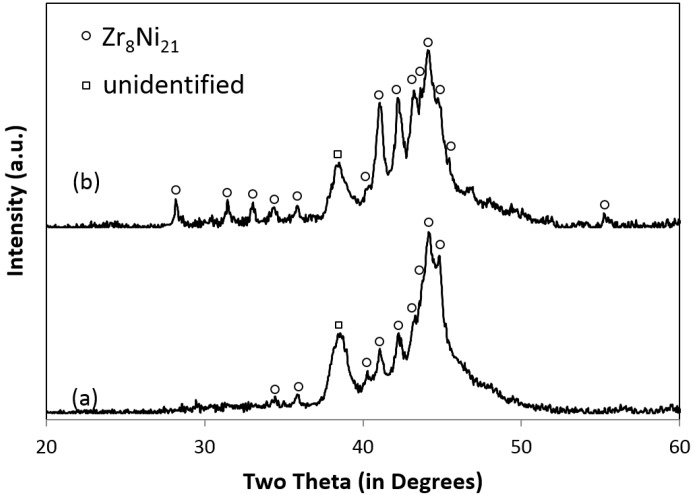
XRD patterns using Cu-K*_α_* as the radiation source for Zr_8_N_21_ as-cast (**a**) and after an 8-h annealing at 960 °C in an Ar environment (**b**).

**Figure 2:**
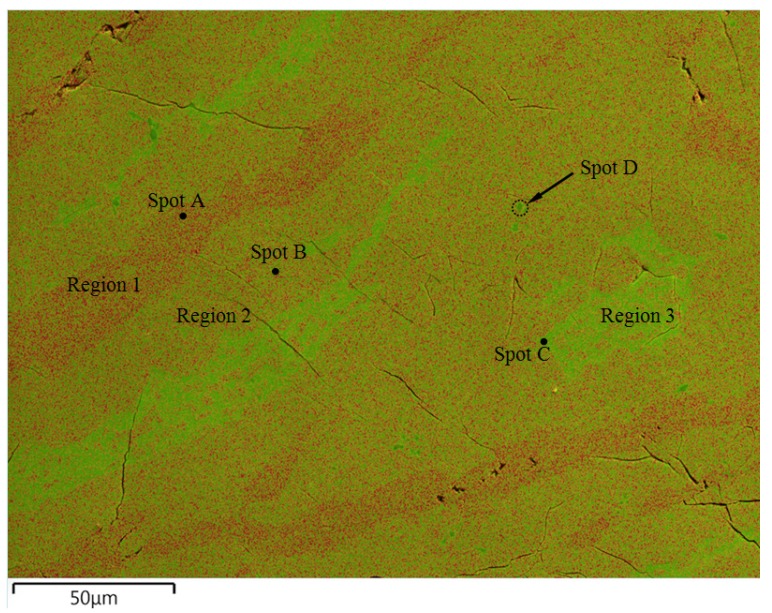
Integrated Zr-L*_α_*_1_ EDS map (green), Ni-K*_α_*_1_ EDS map (red), and SEM secondary electronic image (gray scale), showing different phases in the sample, including Region 1 (Zr_2_Ni_7_), Region 2 (Zr_8_Ni_21_, the major phase), and Region 3 (Zr_7_Ni_10_).

The chemical composition measurement result obtained by ICP from the annealed alloy showed that the actual atomic ratio of Zr/Ni (27.1%/72.9%) was close to the Zr_8_Ni_21_ design (27.6%/72.4%). According to the XRD measurement results from as-cast and annealed samples (Figure 1), most significant peaks were attributed to the Zr_8_Ni_21_ phase, thus indicating that Zr_8_Ni_21_ is the dominant phase of the microstructure. However, there were two broad peaks shown on the XRD pattern, e.g., at 38.4° and 44.6°, which suggested the presence of other phase(s) in addition to Zr_8_Ni_21_. In the previous paper, EDS measurements collected from different spots were used to evaluate phases of the material [11]. Based on the EDS results, it was claimed that there were three phases corresponding to the Zr_8_Ni_21_ phase, a mix of the Zr_8_Ni_21_ and ZrNi phases, and a mix of the Zr_8_Ni_21_ and ZrNi_5_ phases. However, the reason such mixed-phase regions presented was not clear. To verify and further study the structure, the measurements were carried out again at National Institute of Standards and Technology (NIST) with EDS mapping. A composition map of the sample is shown in Figure 2, where three colors, green, red, and gray, correspond to Zr, Ni, and the SEM secondary electron image (SEI), respectively. Quantitative EDS analysis revealed that the Ni/Zr atomic ratios of the identified phases, Regions 1, 2, and 3, are consistent with the stoichiometries close to Zr_7_Ni_10_, Zr_8_Ni_21,_ and Zr_2_Ni_7_, respectively (Table 1). Occasional inclusions of ZrO_2_ were also found in the alloy (Spot D). These ZrO_2_ inclusions appear to be much lower in average atomic weight, showing darker contrast in the SEM backscattering electron image (Figure 1a in Reference [11]).

**Table 1:** Comparison between the EDS measured Ni-Zr composition ratio and the composition from the corresponding stoichiometric phase.

Phases from Figure 2	EDS Zr at%	EDS Ni at%	Corresponding Ni-Zr phases	Stoichiometric Ni at%
Spot A (Region 1)	22 (±2)	78 (±2)	Zr_2_Ni_7_	77.8
Spot B (Region 2)	27 (±2)	73 (±2)	Zr_8_Ni_21_	72.7
Spot C (Region 3)	40 (±2)	60 (±2)	Zr_7_Ni_10_	58.8
Spot D	93 (±2)	7 (±2)	–	–

**Figure 3:**
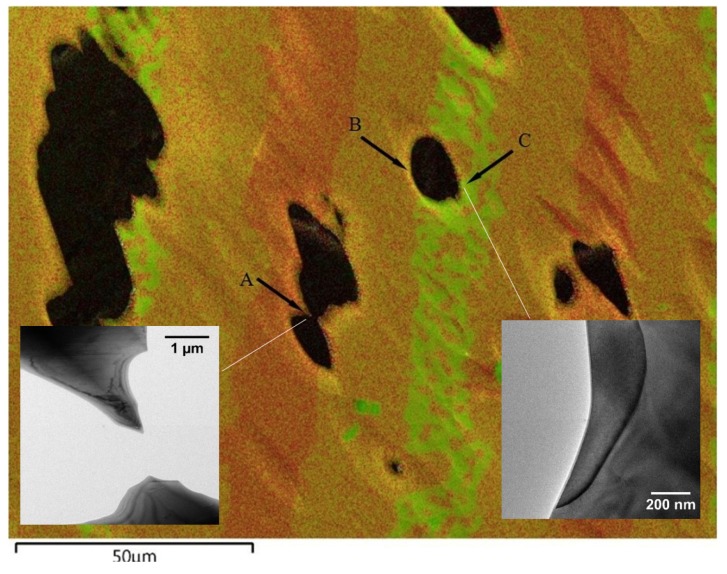
Integrated Zr-L*_α_*_1_ (green) EDS map, Ni-K*_α_*_1_ (red) EDS map, and SEM secondary electronic image (gray scale) obtained from thinned regions of a TEM sample used for diffraction identification of phases.

**Figure 4:**
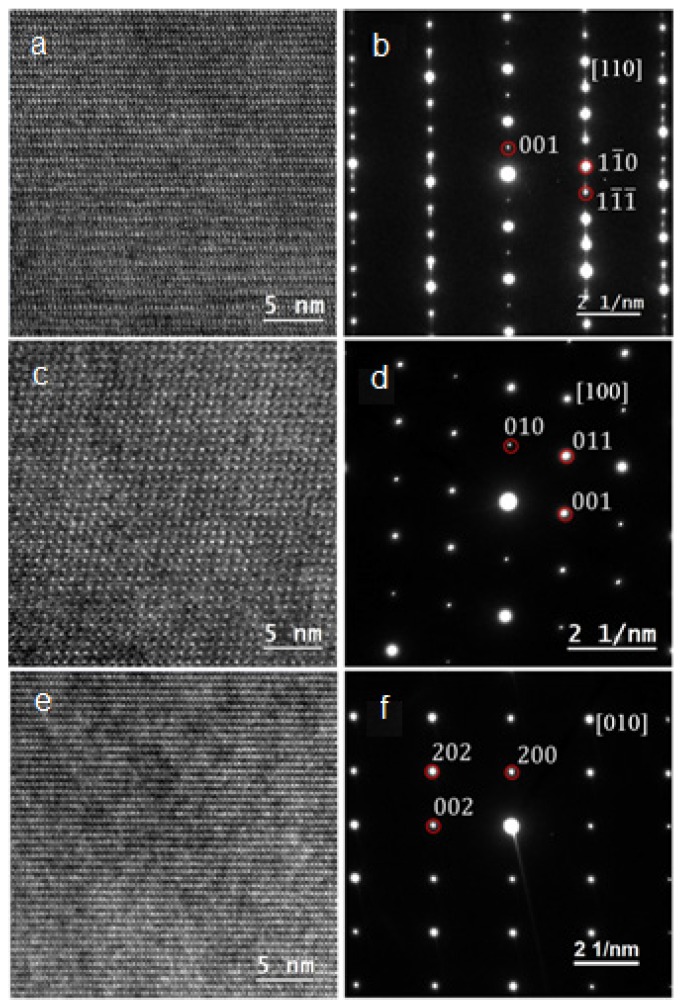
High-resolution TEM images (**a**, **c** and **e**) and corresponding selected area diffraction patterns (**b**, **d** and **f**) obtained from areas A, B and C in Figure 2, respectively. With the help of single crystal diffraction simulations and indexing (red circles), the structures of areas A, B and C are identified as Zr_2_Ni_7_, Zr_8_Ni_21_ and Zr_7_Ni_10_, respectively.

Further confirmation of the phases came from the crystallographic analysis of the phases by electron diffraction from the TEM sample. Before being examined by TEM, SEM/EDS mapping was obtained for the TEM sample, which served as a road map for TEM data collection. For example, in Figure 3, areas A, B, and C are electron-transparent thin areas and are located in a Ni-rich region, a major Zr_8_Ni_21_ phase region, and a Zr-rich region, respectively. Typical bright field TEM images taken from the thin areas are shown in the insets of Figure 3. High resolution TEM images and selected area electron diffraction (SAED) patterns were also taken from these areas, as shown in Figure 4. Indexing of the patterns and comparing with simulations [30,31,32] demonstrated that the Zr-rich phase (green color) has an orthorhombic Zr_7_Ni_10_ structure, while the phase of the highest volume fraction (orange color) has a triclinic Zr_8_Ni_21_ structure, which is consistent with SEM/EDS analysis.

**Figure 5:**
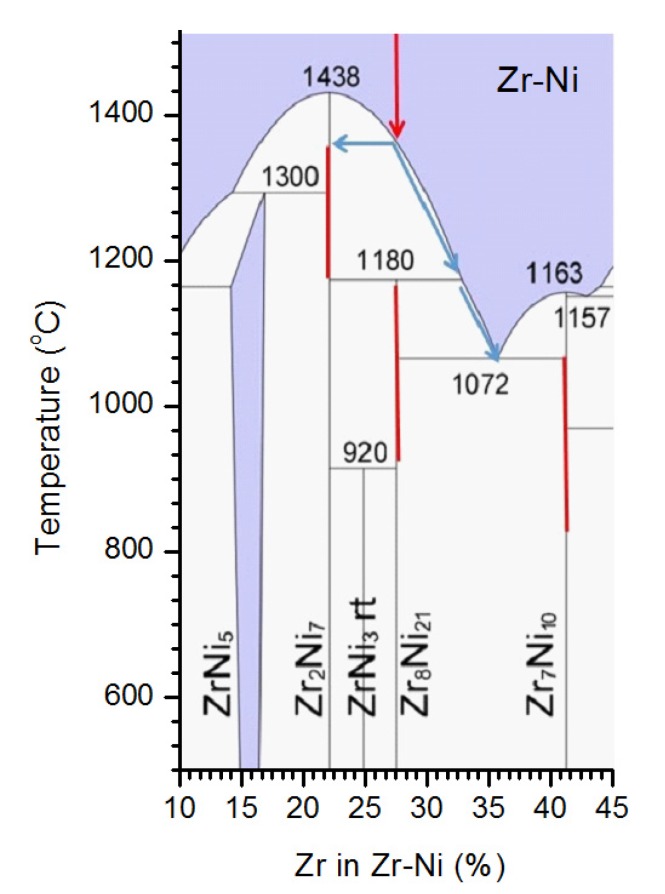
Ni-Zr phase diagram showing formation of the observed phases during continuous cooling of the Zr_8_Ni_21_ melt.

Such phase distribution in this Zr-Ni alloy material can be well explained by the Zr-Ni phase diagram with the assumption that annealing of the alloy was not sufficient to eliminate the cast structure (otherwise a single-phase Zr_8_Ni_21_ structure would be observed) (Figure 5). During the casting process of the sample with a Zr_8_Ni_21_ composition, the Zr_2_Ni_7_ phase is first to crystallize from the melt during cooling down at about 1370 °C. Dendrites of Zr_2_Ni_7_ nucleate and grow until the temperature reaches 1180 °C. At this point, the peritectic reaction between solid Zr_2_Ni_7_ and liquid is attempted; the reaction results in the formation of the Zr_8_Ni_21_ phase that envelops the Zr_2_Ni_7_ dendrites. The peritectic reaction is diffusion-limited, and the continuous cooling may not allow enough time for the reaction to be completed. Thus, the remaining liquid, now enriched in Ni, experiences a eutectic reaction at a temperature below 1072 °C, which results in the formation of a two-phase mixture of Zr_8_Ni_21_ and Zr_7_Ni_10_. With this description the observed microstructure and phases are understood as: Region A, remnants of the first-to-form Zr_2_Ni_7_ phase; Region B, Zr_8_Ni_21_ formed by peritectic reaction; and Region C (of two phases), a product of eutectic reaction L → Zr_8_Ni_21_ + Zr_7_Ni_10_. Judging from its average stoichiometry (59.9% of Ni), Region C is composed of 92% Zr_7_Ni_10_ (58.8% of Ni) and 8% Zr_8_Ni_21_ (72.7% of Ni).

### 3.2. Defects and Crystallography of the Zr_2_Ni_7_ Phase

**Figure 6:**
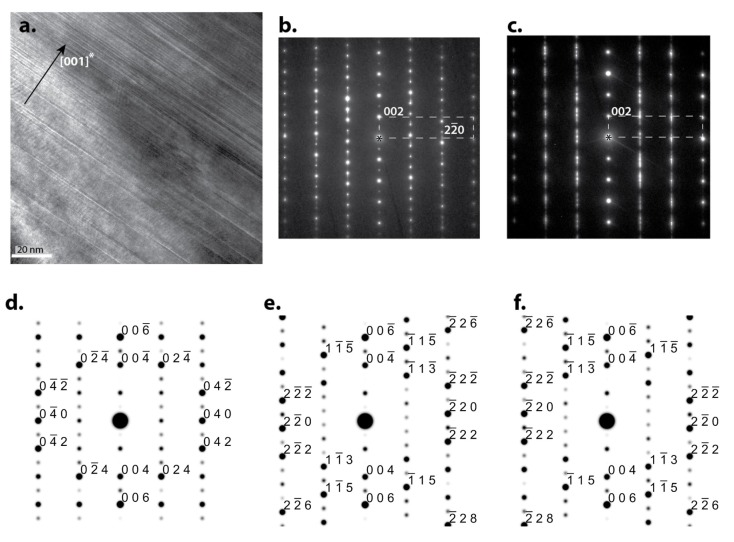
Bright field TEM micrograph of the Zr_2_Ni_7_ phase showing parallel planar defects (**a**) and corresponding SAED patterns (**b**,**c**) showing that the defects’ plane is (001) of the Zr_2_Ni_7_ phase with the simulated diffraction patterns along [100] (**d**), [110] (**e**) and [$\bar{11}0$] (**f**) directions.

While a good agreement among EDS/SEM/TEM measurements and the Ni-Zr phase diagram was established, certain unreported structural features of the Zr_2_Ni_7_ phase have warranted further TEM investigation. Figure 6a shows the bright field image from the region identified by EDS as the Zr_2_Ni_7_ phase. The image shows a high density of planar defects according to SAED patterns. Figure 6b,c indicate the defect planes are (001) of Zr_2_Ni_7_. A SAED pattern forms a region with a lower density of defects in Figure 6b and shows that it can be indexed as Zr_2_Ni_7_ in the [110] zone axis, which is confirmed by comparing it with the simulated [110] patterns, Figure 6e, using a structural model of Eshelman *et al*. [24]. However, SAED from regions with a higher density of defects, Figure 6b, shows a dense distribution of reflection and intensity streaks along the 0 *kl* rows. The reflections can be explained by the overlapping of SAED patterns from three orientations of Zr_2_Ni_7_, [100], [110], and [$\bar{11}$0]; the simulated SAED patterns of these zone axes are shown in Figure 6d–f. From this analysis the observed planar defects can be interpreted as interfaces between 60° rotational (around [001]*) domains of Zr_2_Ni_7_, as well as stacking faults that interrupt the long range ordering sequence.

**Figure 7:**
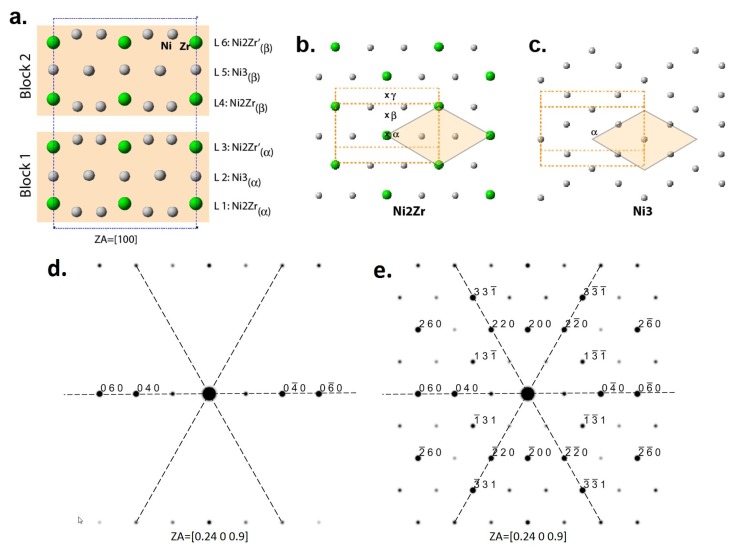
A projection of the Zr_2_Ni_7_ structure in the [100] direction (**a**), projections of the layers from (**a**) with an outlined pseudo-hexagonal unit cell (**b**,**c**) and simulated SAED patterns with the electron beam’s direction normal to the layers’ planes (ZA = [0.24 0 0.9]) for very thin (1 nm) (**d**) and thicker (10 nm) (**e**) Zr_2_Ni_7_ crystals.

The reason for the copious formation of these rotational domains is the pseudo-hexagonal nature of the monoclinic Zr_2_Ni_7_ structure. Figure 7a shows a projection of the structure in the [100] direction; the structure can be described as a stacking of two types of layers with compositions of Ni_2_Zr and Ni_3_. The structure can be subdivided into two very similar blocks consisting of two Ni_2_Zr and one Ni_3_ layers. Figure 7b,c show projections of the layers with an outlined two-dimensional unit cell, from which the pseudo-hexagonal close-packed arrangement of atoms is evident. The hexagonal nature of the layers is not clear from the usual SAED taken with the electron beam’s direction normal to the layers’ planes (ZA = [0.24 0 0.9]) as shown in Figure 7d, but when simulations are done for very thin crystal that allow intersection of intensity rows with the Ewald sphere, the pseudo-hexagonal symmetry becomes evident (Figure 7e). However, the Ni_2_Zr layers, which have identical structural projections, are subdivided into two variants differentiated by the deviation of Zr and Ni from the medial plane: L1 and L4 with Zr upward and L3 and L6 with Ni upward (Figure 7a).

Due to the close-packed (CP) arrangement of atoms within the layers, there are three CP positions, *α*, *β*, and *γ*, for the hard-sphere close-packed staking of these layers in the direction normal to the layers’ planes. In the Zr_2_Ni_7_ structure, the layers within a block are in the same CP position (e.g., *α* for Block 1), but the blocks are shifted to the next CP position (e.g., *β* for Block 2). Thus, the Zr_2_Ni_7_ structure can be described as the following sequence: Ni_2_Zr(*α*)-Ni_3_(*α*)-Ni_2_Zr’(*α*)-Ni_2_Zr(*β*)-Ni_3_(*β*)-Ni_2_Zr’(*β*). Mistakes in 60° rotation of the pseudo-hexagonal layer change the direction of the distortion result in a monoclinic rotational variant with low-energy interdomain (001) interface. Mistakes in the selection of CP position (e.g., *γ* instead of *β*) lead to the formation of low-energy staking faults.

**Figure 8:**
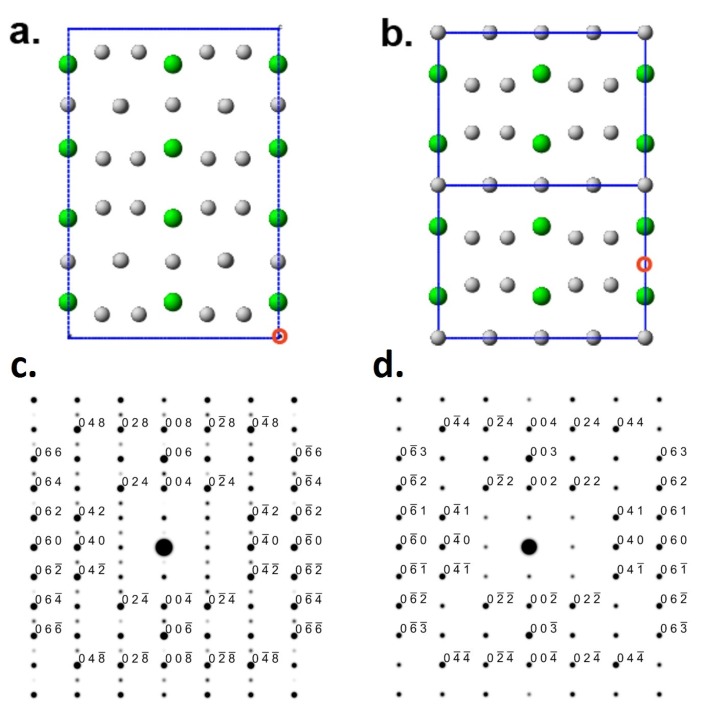
Projection of the Zr_2_Ni_7_ structure in the [100] direction for a structural model of Eshelman [24] and Parthe [27] (**a**), a projection of the layers from (**a**) with an outlined pseudo-hexagonal unit cell (**b**) and simulated SAED patterns with the electron beam’s direction normal to the layers’ planes (ZA = [0.24 0 0.9]) for very thin (1 nm) (**c**) and thicker (10 nm) (**d**) Zr_2_Ni_7_ crystals.

SAED patterns in Figure 6b,c show a significant difference in the intensity of (00*l*) reflections with odd *l*; in some samples' locations, the (00*l*) reflections are completely lacking. These variations can be understood as the following: according to the structural model of Eshelman [24], the structural blocks shown in Figure 7a are very similar but not identical, thus the periodicity *c* = 1.2193 nm. In the analysis by Parthe and Lemair [33], by allowing small changes in the point positions of less than 0.5 nm, a unit-cell transformation can be made which leads to a smaller monoclinic unit cell with *c* = 0.6307 nm. In this structure, Blocks 1 and 2 are identical (Figure 8a,b), but the diffraction is lacking odd (00*l*), or (002) become a new structure (001) (Figure 8c,d).

### 3.3. Software Simulation of Zr_2_Ni_7_ Fine Crystallites

**Figure 9:**
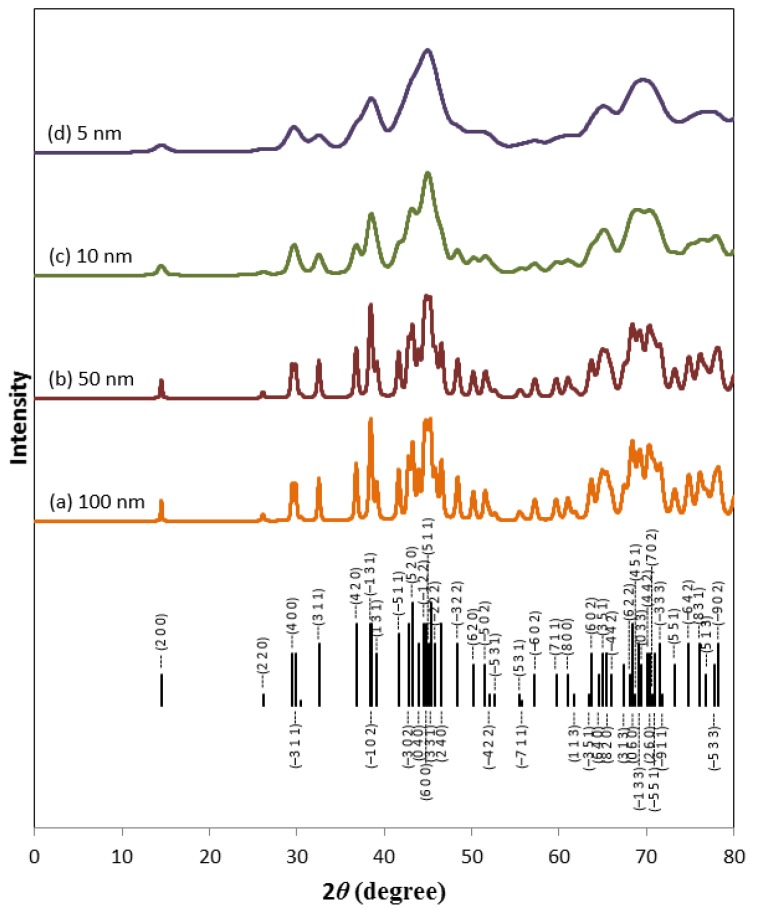
Simulated XRD profile assuming 50% Gaussian and 50% Lorentzian distribution of un-strained crystallite size of 1000 (**a**); 500 (**b**); 100 (**c**); and 50 Å (**d**). The reflections from the Zr_2_Ni_7_ phase are labeled at the bottom. Peak positions and intensities are from PDF 00-026-1291.

From the TEM study of the fine structure of the Zr_2_Ni_7_ phase, it is concluded that the crystallite size of this phase is very small. According to the XRD profile of Zr_2_Ni_7_, it is rather complicated due to its monoclinic nature unlike what is seen in the experimental XRD pattern. However, it is possible for several peaks to overlap as the crystallite size becomes smaller. In order to estimate the crystallite size of Zr_2_Ni_7_, a series of simulated XRD patterns for Zr_2_Ni_7_ with various crystallite sizes under the assumption of 50% Gaussian and 50% Lorentzian distributions are built and shown in Figure 9. As the crystallite size reduces, the XRD peaks become broader and start to overlap. When the crystallite size is below 5 nm, two board peaks centered at around 38.1° and 44.3° are observed. We therefore conclude that those two broad peaks observed in the XRD pattern of the Zr_8_Ni_21_ alloy are from the nano-sized Zr_2_Ni_7_ phase formed by planar defects.

## 4. Conclusions

The microstructure of an annealed alloy of Zr_8_Ni_21_ composition consists of three phases, Zr_8_Ni_21_, Zr_2_Ni_7,_ and Zr_7_Ni_10_. Annealing at 960 °C, which was intended to convert a cast structure into a single-phase Zr_8_Ni_21_ structure, was clearly insufficient. Distribution of the phases and their morphology can be understood with the help of a Zr-Ni phase diagram and is formed by several reactions in the following sequence: (1) L → Zr_2_Ni_7_ + L’; (2) peritectic Zr_2_Ni_7_ + L’ → Zr_2_Ni_7_ + Zr_8_Ni_21_ + L”; (3) eutectic L” → Zr_8_Ni_21_ + Zr_7_Ni_10_. TEM and crystallographic analysis of the Zr_2_Ni_7_ phase show a high density of planar (001) defects, which were explained as low-energy boundaries between 60° rotational variants and stacking faults of near-hexagonal (001) planes. The crystallographic features can be understood as arising from the pseudo-hexagonal nature of Zr_2_Ni_7_, which can be seen as a stacking of near-hexagonal layers with the following sequence: Ni_2_Zr(*α*)-Ni_3_(*α*)-Ni_2_Zr’(*α*)-Ni_2_Zr(*β*)-Ni_3_(*β*)-Ni_2_Zr’(*β*). This highly defective structure of Zr_2_Ni_7_ contributes to those two broad peaks observed in XRD analysis.
